# Supplementary material for: The role of BST4 in the pyrenoid of Chlamydomonas reinhardtii
Source: bioRxiv. 2023 Nov 17:2023.06.15.545204. Preprint. [Version 2] doi: 10.1101/2023.06.15.545204 (PMC10680556; doi:10.1101/2023.06.15.545204)
Supplement: 2 [file NIHPP2023.06.15.545204V2-supplement-1.pdf]

bioRxiv preprint doi: https://doi.org/10.1101/2023.06.15.545204; this version posted November 17, 2023. The copyright holder for this preprint (which was not certified by peer review) is the author/funder, who has granted bioRxiv a license to display the preprint in perpetuity. It is made available under aCC-BY 4.0 International license.

**0.04% CO_2**							
		**Days 0-3**			**Days 0 to 5**		
	Strain	Mean	SE	*P(T<=t)* two-tail	Mean	SE	*P(T<=t)* two-tail
SGR (μ h^{-1})	WT	0.0389	0.0007		0.0241	0.0010	
	bst4	0.0402	0.0003	0.1652	0.0257	0.0001	0.2036
Doubling time (h)	WT	17.8462	0.3380		28.8436	1.2467	
	bst4	17.2379	0.1255	0.1669	26.9798	0.1491	0.2119
**3% CO_2**							
		**Days 0-2**			**Days 0-3**		
	Strain	Mean	SE	*P(T<=t)* two-tail	Mean	SE	*P(T<=t)* two-tail
SGR (μ h^{-1})	WT	0.0679	0.0013		0.0456	0.0011	
	bst4	0.0682	0.0010	0.8626	0.0455	0.0003	0.9436
Doubling time (h)	WT	10.2145	0.1930		15.2239	0.3725	
	bst4	10.1669	0.1510	0.8555	15.2353	0.0878	0.9777

Supplemental Table 1 compares specific growth rates (μ h^{-1}) and cell doubling times of WT and *bst4* strains (n=3) during liquid growth assays at 0.04 and 3 % CO_2 (+/- 2 ppm).
